# Interaction Research on the Antiviral Molecule Dufulin Targeting on Southern Rice Black Streaked Dwarf Virus P9-1 Nonstructural Protein

**DOI:** 10.3390/v7031454

**Published:** 2015-03-23

**Authors:** Zhenchao Wang, Xiangyang Li, Wenli Wang, Weiying Zhang, Lu Yu, Deyu Hu, Baoan Song

**Affiliations:** State Key Laboratory Breeding Base of Green Pesticide and Agricultural Bioengineering/Key Laboratory of Green Pesticide and Agricultural Bioengineering, Ministry of Education, Guizhou University, Guiyang 550025, China; E-Mails: wzc.4884@163.com (Z.W.); xiangyang li83@126.com (X.L.); wangwenli0208@163.com (W.W.); zwy105@163.com (W.Z.); yuji570@163.com (L.Y.); fcc.dyhu@gzu.edu.cn (D.H)

**Keywords:** interaction research, complete sequence, antiviral molecule, dufulin, nonstructural P9-1 protein, mutant protein, microscale thermophoresis, fluorescence titration

## Abstract

Southern rice black streaked dwarf virus (SRBSDV) causes severe harm to rice production. Unfortunately, studies on effective antiviral drugs against SRBSDV and interaction mechanism of antiviral molecule targeting on SRBSDV have not been reported. This study found dufulin (DFL), an ideal anti-SRBSDV molecule, and investigated the interactions of DFL targeting on the nonstructural protein P9-1. The biological sequence information and bonding characterization of DFL to four kinds of P9-1 protein were described with fluorescence titration (FT) and microscale thermophoresis (MST) assays. The sequence analysis indicated that P9-1 had highly-conserved *C*- and *N*-terminal amino acid residues and a hypervariable region that differed from 131 aa to 160 aa. Consequently, wild-type (WT-His-P9-1), 23 *C*-terminal residues truncated (TR-ΔC23-His-P9-1), 6 *N*-terminal residues truncated (TR-ΔN6-His-P9-1), and Ser138 site-directed (MU-138-His-P9-1) mutant proteins were expressed. The FT and MST assay results indicated that DFL bounded to WT-His-P9-1 with micromole affinity and the 23 *C*-terminal amino acids were the potential targeting site. This system, which combines a complete sequence analysis, mutant protein expression, and binding action evaluating system, could further advance the understanding of the interaction abilities between antiviral drugs and their targets.

## 1. Introduction

Plant-infecting reoviruses are classified into three genera, namely *Phytoreovirus*, *Oryzavirus* and *Fijivirus*. Fijivirus species containing 10 segments, with an aggregate genome size larger than in other plant reovirus genera are distributed worldwide, and some of them cause serious diseases [1,2]. Southern rice black streaked dwarf virus (SRBSDV) transmitted by white-backed planthopper (*Sogatella furcifera* Horváth) vectors in a persistent propagative manner is a member of the genus *Fijivirus* of the *Reoviridae* family; it is a non-enveloped double-stranded RNA virus that has caused significant losses of grain yields in parts of Asia, including Vietnam, Japan, and China [3,4,5,6,7]. However, no effective anti-SRBSDV drugs have been developed to prevent and control SRBSDV to ensure rice production.

SRBSDV consists of icosahedral particles and has 10 segments, designated as S1 to S10 according to their size that ranges from approximately 4.5 kb to 1.4 kb [3,4,7,8]. A comparison of the different genomic segments of SRBSDV with those of their counterparts in rice black streaked dwarf virus (RBSDV) suggests that SRBSDV encodes at least six putative structural proteins (P1, P2, P3, P4, P8, and P10) and five putative nonstructural proteins (P6, P7-1, P7-2, P9-1, and P9-2) [9]. Among the putative structural proteins encoded by SRBSDV, P1 and P2 are a putative RNA-dependent RNA polymerase and a core protein, respectively; P4 and P3 are an outer-shell B-spike protein and a putative capping enzyme, respectively [10,11]; and P8 and P10 are a putative core protein and a major outer capsid protein, respectively [12,13]. Among the putative nonstructural proteins encoded by SRBSDV, P6 is a viral RNA-silencing suppressor [14], and P7-1 has the intrinsic ability to self-interact to form tubules in non-host insect cells [15].

The most important nonstructural protein in SRBSDV replication may be the nonstructural protein P9-1, which is a major constituent of the viroplasm (Vps) and has 77% amino acid identity to its counterpart RBSDV-P9-1 [10]. For the genus *Fijivirus*, P9-1 performs an important function in the early stages of the virus life cycle by forming intracellular Vps, which serves as sites for virus replication and assembly [16]. In cultured insect vector cells, the knockdown of SRBSDV-P9-1 expression caused by RNA interference strongly inhibits Vps formation and viral infection [17]. Meanwhile, as evidenced by electron and confocal microscopy, P5, P6, and P9-1 are collectively required for the genesis and maturation of the filamentous and granular Vps matrix induced by SRBSDV infection [18]. These reports suggest that the gene for SRBSDV P9-1, which is the functional ortholog of RBSDV P9-1 based on homology searches, may be a critical target to suppress SRBSDV proliferation in infected rice plants.

Based on our previous research, dufulin (DFL), which displayed the highest inhibitory activity against SRBSDV, was developed as an anti-SRBSDV molecule [19]. However, the function, if any, that DFL performs in the antiviral mechanism against SRBSDV is unclear. Thus, in view of the importance of the nonstructural viral protein P9-1, a unique interaction mechanism and its implications for DFL’s targeting and binding affinity were investigated.

In this study, complete sequences of P9 of nine isolates from China were obtained. The results indicated the highly conserved regions in the *C* and *N* terminals of P9-1 and the hypervariable region at 131 aa to 160 aa. Wild-type P9-1 protein (WT-His-P9-1), 23 *C*-terminal residues truncated (TR-ΔC23-His-P9-1), 6 *N*-terminal residues truncated (TR-ΔN6-His-P9-1), and Ser138 site-directed (MU-138-His-P9-1) mutant proteins were expressed successfully based on the bioinformatic analysis results of the complete sequences. WT-His-P9-1 and the three different mutagenesis nonstructural proteins were subsequently chosen as models for the interaction tests with DFL by fluorescence titration (FT) and microscale thermophoresis (MST) assays, and ningnanmycin (NNM) was used as control. Furthermore, DFL had higher binding affinity than NNM to WT-His-P9-1 with a dissociation constant (*K*_d_) of 3.26 μM by MST and a binding constant (*K*_A_) of 1 × 10^5.06^·M^−1^ by FT. Nevertheless, the P9-1 mutagenesis protein truncated with 23 *C*-terminal residues disrupted the binding ability of DFL with a dissociation constant (*K*_d_) of 70.30 μM and a binding constant (*K*_A_) of 1 × 10^4.47^·M^−1^. In conclusion, DFL had a strong binding affinity to P9-1 to exert an anti-SRBSDV mechanism, and the 23 *C*-terminal amino acids were the binding sites. This system, which combines complete sequence analysis, mutant protein expression, and binding action evaluating platform, could further advance the understanding of the action mechanism between antiviral drugs and their targets.

## 2. Materials and Methods

### 2.1. Samples and RNA Isolation

All of the isolates were collected from rice fields in Libo City (GZLBo), Pingtang City (GZPTang), and Duyun City (GZDYun) of Guizhou Province; Longchuan City (YNLChuan), Zhaotong City (YNZTong), and Yuanjiang City (YNYJiang) of Yunnan Province; Luxi City (JXLXi) of Jiangxi Province; and Jianghua City (HNJHua) of Hunan Province. An artificial inoculation in the seeding stage conducted in a greenhouse was also chosen (GZZCD). All infected leaves were frozen and stored at −80 °C. Total RNAs were extracted from the rice leaves using a Trizol kit (TAKARA, Dalian, China) following the manufacturer’s instructions.

### 2.2. Cloning of the Complete SRBSDV-P9 Gene

Reverse transcription was performed using a cDNA synthesis system (TAKARA, Dalian, China), with P9-C1 primer as reverse primer [20] (Table 1). After mixing the components (*i.e.*, 5 × MLV buffer, dNTP, RNase inhibitor, RNase-free H_2_O, and M-MLV reverse transcriptase), the sample was incubated at 42 °C for 1 h and then heated at 70 °C for 10 min. PCR was performed with *LA*Taq^TM^ DNA polymerase (TAKARA, Dalian, China) at 94 °C for 4 min, followed by 30 cycles of denaturation at 94 °C for 40 s, annealing at 57 °C for 45 s, extension at 72 °C for 105 s, and final extension at 72 °C for 10 min. The PCR products were purified and cloned into the pMD 19-T vector using a T vector kit (TAKARA, Dalian, China) and then transformed in *Escherichia coli* DH5ɑ for sequencing.

**Table 1:** Primers used in this study.

Primer Name	Sequence(5'→3') ^a^
P9-N1	AAGTTTTTTAAGCCTGGAACTGAC
P9-C1	GACATCAGCTGTAAGCCGG
WT-His-P9-1-N2	GGAATTCCATATGGCAGACCTAGAGCGTAGAA
WT-His-P9-1-C2	CCGCTCGAGTCAAACGTCCAATTTAAGTGAAGAA
TR-ΔC23-His-P9-1-N3	GGAATTCCATATGGCAGACCTAGAGCGTAGA
TR-ΔC23-His-P9-1-C3	CCGCTCGAGTCAAAAACGACGATATCTTTT
TR-ΔN6-His-P9-1-N4	GGAATTCCATATGACGTTTGGATCATATA
TR-ΔN6-His-P9-1-C4	CCGCTCGAGTCAAACGTCCAATTTAAGTGAA
Mu-138-His-P9-1-N5	CTTTTTGGTCTTTAGTTGTGGATTCGCTTTCAACGAC
Mu-138-His-P9-1-C5	GTCGTTGAAAGCGAATCCACAACTAAAGACCAAAAAG

^a^ Restriction sites are underlined.

### 2.3. Bioinformatic Analysis of the SRBSDV-P9-1 Sequence

The physicochemical properties of nine complete nucleotide sequences of P9 were predicted using ExPA Syproteomics server [21]. The property analyses of P9-1 protein based on their primary amino acid sequences were predicted. The hydrophilic and hydrophobic properties of the nine P9-1 isolates were analyzed using the ProtScale program [22] of ExPASy, with Hphob/Kyte and Doo little parameter setting. Signal P 4.0 Server [23] was used to predict the signal peptide of P9-1 protein. The prediction of P9-1 protein subcellular location was deduced from WoLF PSORT [24]. The transmembrane helical area and functional domains in P9-1 were predicted using the programs TMHMM Server [25] and SMART5.0 [26].

The secondary structures of protein P9-1 were predicted based on their primary amino acid sequences using SOPAM [27].

Three methods were applied in homology analysis: Recombination analysis with RDP software, multiple-sequence alignments with GeneDoc, and single-nucleotide polymorphism (SNP) analysis with Generous software.

In the multiple-sequence alignments, aside from the 9 P9-1 sequences obtained in the experiments, 11 SRBSDV-P9-1 sequences (GenBank: HM585271.1, HQ394211.1, JQ773428.1, HM998852.1, EU523359.1, KF444269.1, JQ692580.1, KF444267.1, KF494221.1, KF494220.1, HQ731500.1) and 12 RBSDV-P9-1 sequences (GenBank: DQ407917.1, AF536564.2, AF540976.1, KC875238.1, JX421771.1, KC134297.1, AJ297430.1, AJ297429.1, AF459812.1, AY039705.1, AY050487.1, and AY050486.1) of NCBI were downloaded for analysis.

In the SNP analysis, 11 SRBSDV-P9-1 sequences and 12 RBSDV-P9-1 sequences downloaded from NCBI and 9 SRBSDV-P9-1sequences from our sequencing tests were used.

### 2.4. Construction of WT-His-P9-1 and Mutant Plasmids

In P9-1 cloning, first-strand cDNA templates were synthesized using 2 μg of the total RNA of GZLBo with random primers using M-MLV reverse transcriptase (TAKARA, Dalian, China). After mixing the components (*i.e.*, 5 × Prime Script^TM^ buffer, Prime Script^TM^ enzyme mix, and RNase-free H_2_O), the sample was incubated at 37 °C for 15 min and then heated at 85 °C for 5 s. PCR was performed with Prime STAR HS DNA polymerase (TAKARA, Dalian, China) at 98 °C for 5 min, followed by 30 cycles of denaturation at 98 °C for 10 s, annealing at 55 °C for 10 s, extension at 72 °C for 1 min, and final extension at 72 °C for 10 min.

The primers used to amplify the coding region for the expression of WT-His-P9-1, TR-ΔC23-His-P9-1, and TR-ΔN6-His-P9-1 contained restriction sites of *NdeΙ* or *XhoΙ*, *i.e.*, WT-His-P9-1-N2 and WT-His-P9-1-C2 were used to amplify the coding region for WT-His-P9-1; TR-ΔC23-His-P9-1-N3 and TR-ΔC23-His-P9-1-C3 for TR-ΔC23-His-P9-1; and TR-ΔN6-His-P9-1-N4 and TR-ΔN6-His-P9-1-C4 for TR-ΔN6-His-P9-1. After digestion, the PCR-amplified fragments were ligated into *NdeΙ*-*XhoΙ*-digested pET28a vectors, named as pET28a-WT-P9-1, pET28a-ΔC23-P9-1, and pET28a-ΔN6-P9-1. Each constructed plasmid was transformed into *E. coli* DH5ɑ, amplified, and sequenced.

The plasmid pET28a-WT-P9-1 served as template for site-directed PCR. Site-directed mutagenesis was used to replace the single site at Ser138 with Thr138 using the two-step PCR procedure described by Yin *et al.* [28]. PCR was performed with a Prime STAR polymerase kit (TAKARA, Dalian, China) with the primers of Mu-138-His-P9-1-N5 and Mu-138-His-P9-1-C5. After PCR, *DpnΙ* was added to 50 μL of the PCR mixtures and incubated at 37 °C for 1 h. The resulting plasmid was transformed into *E. coli* DH5ɑ, amplified, and sequenced.

### 2.5. Purification of the SRBSDV-P9-1 Proteins

Genetically engineered WT-His-P9-1 and mutagenesis proteins were expressed, into which hexahistidine (His) tags were incorporated. To express the wild-type and mutant forms of P9-1, we transformed each plasmid into *E. coli* BL_21_ (DE_3_) RIL. The *E. coli* BL_21_ (DE_3_) RIL harboring the recombinant plasmid was cultured in LB medium that contains 30 μg kanamucin/mL at 37 °C until the OD_600_ reached 0.8. Protein expression was induced at 16 °C by adding IPTG at 1 mM for 16 h. Bacteria were harvested by centrifugation at 12,000 × g for 10 min at 4 °C. The pellets were suspended in a buffer that contains 150 mM NaCl, 20 mM imidazole, 20 mM NaH_2_PO_4_·2H_2_O, 30 mM Na_2_HPO_4_·12H_2_O, and 1 ‰ beta-mercaptoethanol. The suspension was sonicated (Noise Isolating Tamber, Scientz, USA) for 35 min in an ice bath and then centrifuged at 12,000 × g for 30 min at 4 °C to remove cell debris. The protein was purified from the supernatant using Ni–NTA kit (ACTA, Purifier TM UPC10, Pittsburgh, PA, USA). The Ni-NTA column was washed with 20 mM imidazole wash buffer (20 mM NaH_2_PO_4_, 150 mM NaCl, 20 mM imidazole, and 30 mM Na_2_HPO_4_, Ph 7.4), and the proteins were eluted with 250 mM imidazole elution buffer (50 mM NaH_2_PO_4_, 300 mM NaCl, and 250 mM imidazole, pH7.4). The protein was desalinated into a storage buffer (40 mM Na_2_HPO_4_, 10 mM NaH_2_PO_4_, and 300 mM NaCl, pH 7.4), and then the sample was dispensed into aliquots and stored at −70 °C until use.

The expressed proteins of WT-His-P9-1, TR-ΔC23-His-P9-1, TR-ΔN6-His-P9-1, and MU-138-His-P9-1 were initially assayed by Coomassie brilliant blue method in a small-scale experiment, in which the final concentration was 20 μM. The target proteins were confirmed by 12% sodium dodecylsulfate polyacrylamide gel electrophoresis (SDS-PAGE).

### 2.6. Fluorescence Spectra Studies

The chemical structures of DFL and NNM are shown in Supplementary Figure 1. Fluorescence spectra were obtained with FluoroMax^®^-4 and FluoroMax^®^-4P (HORIBA Scientific, Paris, France). The fluorescence quenching spectra were recorded at 298 K from 285 nm to 400 nm at 275 nm excitation wavelength. The excitation and emission band widths were both 10 nm. The concentration of WT-His-P9-1 and mutant proteins for each run was fixed at 20 μM, and the antiviral DFL concentrations were *C*_DFL_: 1–11: 0, 2.0, 4.0, 6.0, 8.0, 10.0, 12.0, 14.0, 16.0, 18.0, 20.0 μM, and the antiviral NNM concentrations were *C*_NNM_: 1–11: 0, 4.0, 8.0, 12.0, 16.0, 20.0, 24.0, 28.0, 32.0, 36.0, 40.0 μM.

Spectrometry is one of the various methods used to evaluate protein–drug interactions. The intrinsic fluorescence of WT-His-P9-1 was mostly contributed by both Phe and Tyr (Supplementary File 1). To test the binding ability of DFL to WT-His-P9-1, we employed FT assay, with NNM as contrast. Fluorescence quenching could be dynamic, resulting from collisional encounters between the fluorophore (protein) and the quencher, or static, resulting from the formation of a ground-state complex between the fluorophore and the quencher [29]. To verify the type of fluorescence quenching, the Stern–Volmer equation was applied [30,31,32], as shown in Equation (1):

*F*_0_/*F* = 1 + *K*_q_*τ*_0_ [*Q*] = 1 + *K*_sv_ [*Q*] (1)

where *F*_0_ and *F* are the fluorescence intensities without and with the quencher, respectively; *K*_q_ and *K*_sv_ are the quenching rate constant of the biomolecule and the Stern–Volmer dynamic quenching constant, respectively; [*Q*] is the quencher concentration; and *τ*_0_ (1 × 10^−8^ s) is the average lifetime of BSA without the quencher. When molecules bind independently to a set of equivalent sites on a macromolecule, *K*_A_ and the number of binding sites (*n*) could be obtained from the Hill equation [33]:

*lg* (*F*_0_ − *F*)/*F = lgK*_A_*+ n lg*[*Q*] (2)

### 2.7. MST Studies

The WT-His-P9-1 and mutant proteins were labeled for MST using Monolith NT protein labeling kit Red (Nano Temper Technologies, München, Germany), as recommended by the manufacturer. The concentrations of DFL and NNM binding reactions were carried out using Monolith NT.115 (Nano Temper Technologies, München, Germany). The binding data were analyzed using GraphPad Prism to estimate the *K*_d_ values.

## 3. Results

### 3.1. Bioinformatic Analysis of SRBSDV-P9-1 Sequence

The complete nucleotide and amino acid sequences of the genomic segment P9 of nine isolates from Libo City (GZLBo, GenBank: KJ513450), Pingtang City (GZPTang, GenBank: KJ513451), and Duyun City (GZDYun, GenBank: KJ513449) of Guizhou Province; Longchuan City (YNLChuan, GenBank: KJ513453), Zhaotong City (YNZTong, GenBank: KJ513455), and Yuanjiang City (YNYJiang, GenBank: KJ513454) of Yunnan Province; Luxi City (JXLXi, GenBank: KJ513457) of Jiangxi Province; and Jianghua City (HNJHua, GenBank: KJ513456) of Hunan Province were analyzed. An artificial inoculation in the seeding stage conducted in a greenhouse in Guiyang City was also selected (GZZCD, GenBank: KJ513452).

The physicochemical properties of the nine complete nucleotide sequences of SRBSDV-P9 were initially predicted. According to the results of the nine complete nucleotide sequences, SRBSDV-P9 consisted of 1900 base pairs (bp), similar to other reported SRBSDV isolates downloaded from NCBI. The SRBSDV-P9 genome contained two long, non-overlapping open reading frames (ORFs) (P9-1 and P9-2), which encoded polypeptides of 39.9 kDa (P9-1, from 53 nt to 1096 nt) and 24.2 kDa (P9-2, from 1160 nt to 1789 nt). The GC contents of the nine SRBSDV-P9 isolates, namely, GZLBo, GZPTang, GZDYun, GZZCD, YNLChuan, YNZTong, YNYJiang, JXLXi, and HNJHua, were 35.53%, 34.63%, 34.74%, 34.53%, 34.58%, 35.27%, 34.63%, 34.58%, and 34.74%, respectively, ranging from 34.53% to 35.53%.

**Figure 1:**
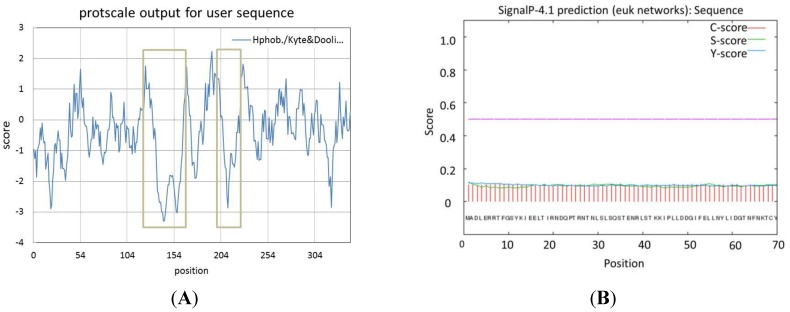

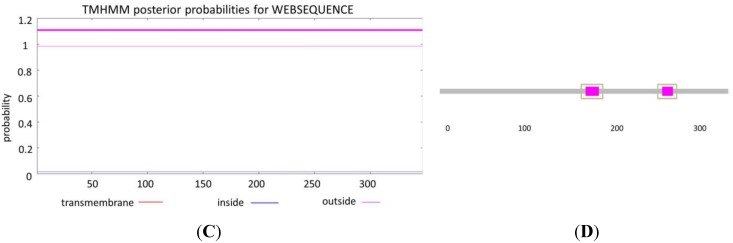
Bioinformatic analysis of GZDYun-P9-1. (**A**) Hydrophilic regions shown within the box; (**B**) signal peptide analysis; (**C**) transmembrane structures in P9-1 protein deduced by TMHMM Server program; and (**D**) low-complexity regions within the box deduced by SMART5.0.

As revealed in RBSDV counterparts, P9-2 was not detected in RBSDV-infected plants and insects, whereas P9-1 accumulates in intracellular Vps, functioning as a probable component of Vps and therefore performing an important function in Vps formation and viral morphogenesis [10]. Thus, the properties of SRBSDV-P9-1 protein were predicted by bioinformatic analysis method based on its important function. The nine SRBSDV-P9-1 proteins have similar characteristics; hence, GZDYun-P9-1 of the GZDYun isolate was introduced as representative. Two hydrophilic regions located at 125–170 and 210–230 aa existed in GZDYun-P9-1 (Figure 1A). Signal peptides did not exist in GZDYun-P9-1, as analyzed by Signal P 4.0 procedure (Figure 1B). The WoLF PSORT analysis results indicated that the subcellular location of GZDYun-P9-1 was the cell nuclei. No transmembrane structures were found in GZDYun-P9-1, as deduced by TMHMM Server program (Figure 1C). The SMART5.0 results proved that two low-complexity regions located at 176–190 and 268–279 aa existed in GZDYun-P9-1 (Figure 1D). Furthermore, the helix, sheet, turn, and coil of the secondary structures of protein P9-1 were approximately 41%, 18%, 6%, and 35%, respectively.

### 3.2. Recombination and Multiple-Sequence Alignment Analysis of the SRBSDV-P9-1 Sequence

Recombination analysis revealed that no recombinant phenomenon occurred in the nine P9-1 sequences.

**Figure 2:**
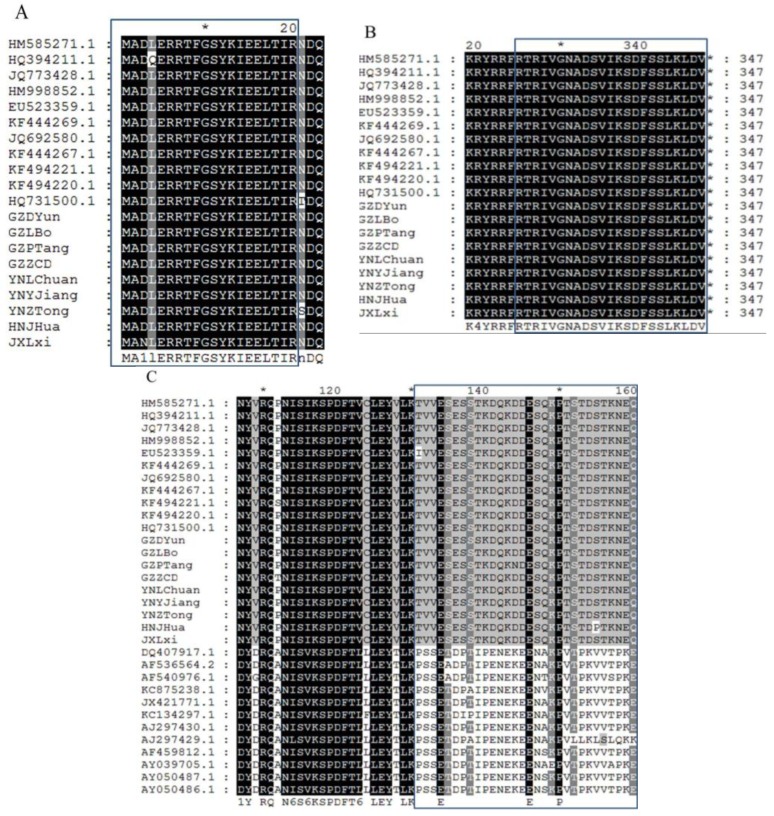
Alignments of SRBSDV-P9-1 and RBSDV-P9-1 by Gene Doc software. (**A**) Highly conserved *N-*terminal residues deduced by the alignment of 11 SRBSDV-P9-1 sequences downloaded from NCBI (GenBank: HM585271.1, HQ394211.1, JQ773428.1, HM998852.1, EU523359.1, KF444269.1, JQ692580.1, KF444267.1, KF494221.1, KF494220.1, HQ731500.1) and nine sequences in the sequencing tests (GenBank: GZDYun, GZLBo, GZPTang, GZDYun, YNLChuan, YNZTong, YNYJiang, JXLXi, HNJHua); (**B**) highly conserved *C*-terminal residues deduced by the alignment of 11 SRBSDV-P9-1 sequences downloaded from NCBI (GenBank: HM585271.1, HQ394211.1, JQ773428.1, HM998852.1, EU523359.1, KF444269.1, JQ692580.1, KF444267.1, KF494221.1, KF494220.1, HQ731500.1) and nine SRBSDV-P9-1 sequences in the sequencing tests (GenBank: GZDYun, GZLBo, GZPTang, GZDYun, YNLChuan, YNZTong, YNYJiang, JXLXi, HNJHua); (**C**) highly hypervariable region from 131 aa to 160 aa deduced by the alignment of 11 SRBSDV-P9-1 sequences downloaded from NCBI (GenBank: HM585271.1, HQ394211.1, JQ773428.1, HM998852.1, EU523359.1, KF444269.1, JQ692580.1, KF444267.1, KF494221.1, KF494220.1, HQ731500.1), 9 SRBSDV-P9-1 sequences in the sequencing tests (GenBank: GZDYun, GZLBo, GZPTang, GZDYun, YNLChuan, YNZTong, YNYJiang, JXLXi, HNJHua), and 12 RBSDV-P9-1 sequences downloaded from NCBI (GenBank: DQ407917.1, AF536564.2, AF540976.1, KC875238.1, JX421771.1, KC134297.1, AJ297430.1, AJ297429.1, AF459812.1, AY039705.1, AY050487.1, AY050486.1).

The sequence comparison analysis of the nine isolates from China and the 11 SRBSDV-P9-1 sequences, which had been reported previously on NCBI, revealed that SRBSDV-P9-1 had a high degree of identity, especially in the *N*- and *C*-terminal residues. In front of the *N*-terminal residues, the amino acids of the 20 SRBSDV-P9-1 sequences were almost the same in the following order: M, A, D, L, E, R, R, T, F, G, S, Y, K, I, E, E, L, T, I, R (Figure 2A). Similarly, in the last 23 *C*-terminal residues, the amino acids of the 20 SRBSDV-P9-1 sequences remained highly conserved in the following order: R, T, R, I, V, G, N, A, D, S, V, I, K, S, D, F, S, S, L, K, L, D, V (Figure 2B). A highly hypervariable region from 131 aa to 160 aa was deduced by the alignment of the 11 SRBSDV-P9-1 sequences downloaded from NCBI, nine SRBSDV-P9-1 sequences in our sequencing tests, and 12 RBSDV-P9-1 sequences downloaded from NCBI. The amino acids of SRBSDV-P9-1 protein from 131 aa to 160 aa remained highly conserved and were significantly distinguished from those of RBSDV-P9-1. Almost all amino acids of SRBSDV-P9-1 from 131 aa to 160 aa were in the following order: T, V, V, E, S, E, S, S, T, K, D, Q, K, D, D, E, S, Q, K, P, T, S, T, D, S, T, K, N, E, Q, which is obviously different from RBSDV-P9-1 in the following order: P, S, S, E, T, D, P, T, I, P, E, N, E, K, E, E, N, A, K, P, V, T, P, K, V, V, T, P, K, E.

### 3.3. SNP Analysis of the SRBSDV-P9-1 Sequence

Sequence variants are responsible for the genetic components of individuality, including complex characteristics, such as disease susceptibility and drug response. Most sequence variants are SNPs, in which two alternate bases exist at one position [34,35]. In the 1044 nt of the SRBSDV-P9-1 sequence analyzed, six SNPs were identified (Table 2). Single-base substitutions based on transitions or transversions were classified as follows: Three transitions on the CDS positions of 951, 540, and 384 nt and three transversions on the CDS positions of 898, 415, and 384 nt. Obviously, all six SNPs were considered coding SNPs (cSNP) because of their location on the CDS position. Four of them were classified as synonymous cSNP (sSNP), which did not cause changes in the amino acids on the CDS positions of 951, 540, 384, and 141 nt. However, two SNPs were considered non-synonymous cSNP (nsSNP) on the CDS positions of 898 and 415 nt for causing the P **→** T^898^ and S **→** T^415^ phenomena, respectively.

**Table 2:** SNPs of SRBSDV-S9.

NO.	Amino Acids	CDS Position	Codon
1		951	AAT → AAC
2	P → T	898	CCA → ACA
3		540	TCT → TCC
4	S → T	415	TCT → ACT
5		384	GTC → GTT
6		141	CTC → CTA

### 3.4. Expression and Purification of the SRBSDV-P9-1 Nonstructural Proteins

**Figure 3:**
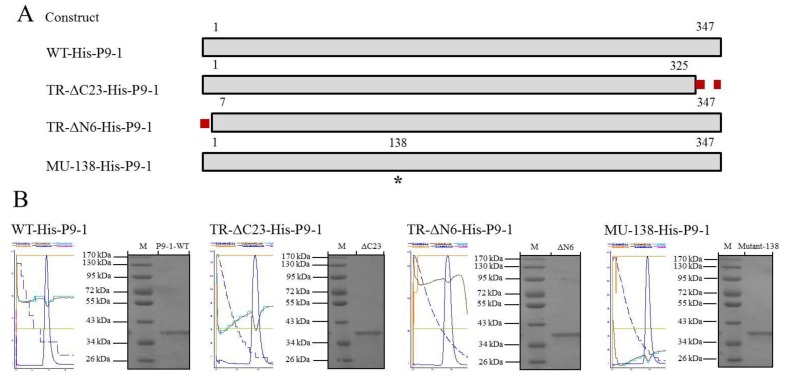
The recombinant plasmid construction and purification of target proteins. (**A**) Schematic illustration of SRBSDV P9-1 protein, which has 347 amino acids. Construct names are indicated in the left blocks to represent the P9-1 region contained in the construct. The discontinuous red lines represent the deleted region. The numbers indicate the relative amino acid positions in P9-1. The asterisk (*) on the 138 residues indicates the site of site-directed mutagenesis; (**B**) expression and purification of the P9-1 protein. The four parts named WT-His-P9-1, TR-ΔC23-His-P9-1, TR-ΔN6-His-P9-1, and MU-138-His-P9-1 represent the desalted proteins. The proteins were separated by 12% SDS–PAGE, with the protein molecular weight standards shown in lane M.

To obtain large amounts of P9-1 protein for biochemical characterization, we cloned full-length SRBSDV-P9-1 gene into pET28a. The above genetically engineered SRBSDV-P9-1 and three mutagenesis proteins were expressed, into which hexahistidine (His) tags were incorporated (Figure 3A). Given that His-tags were short peptides, a series of nonstructural P9-1 proteins including WT-His-P9-1 protein and those truncated from the *C* and *N* terminals, designated as TR-ΔC23-His-P9-1 and TR-ΔN6-His-P9-1, respectively, was constructed. Simultaneously, the amino acid residue of Ser138 was subjected to site-directed mutagenesis to investigate its function and was named MU-138-His-P9-1.

These correct proteins were successfully cloned to the expression host, *E. coli* BL_21_(DE_3_) RIL, for protein expression. After induction, the *N*-terminally 6 × His-tagged recombinant P9-1 proteins were purified with Ni-NTA resin, eluted with 250 mM imidazole elution buffer, and separated by 12% SDS-PAGE. As predicted, SRBSDV-P9-1 contained an ORF, encoded a protein with 347 amino acids, and had a molecular mass of 40.0 kDa. The molecular mass of WT-His-P9-1, TR-ΔC23-His-P9-1, TR-ΔN6-His-P9-1, and MU-138-His-P9-1 were tested in a similar migration approximately at 40.0 kDa as well (Figure 3B).

### 3.5. Binding Properties of DFL and NNM to Wild-Type SRBSDV-P9-1 Nonstructural Protein

**Figure 4:**
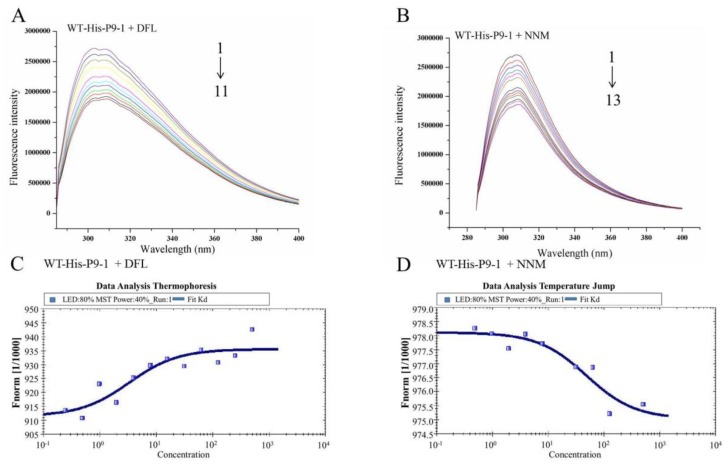
Binding affinity and specificity of DFL and NNM to WT-His-P9-1 protein. (**A**) Fluorescence emission spectra of WT-His-P9-1 in the presence of DFL, *C*_WT-His-P9-1_: 20 μM, *C*_DFL_: 1–11: 0, 2.0, 4.0, 6.0, 8.0, 10.0, 12.0, 14.0, 16.0, 18.0, 20.0 μM. (**B**) Fluorescence emission spectra of WT-His-P9-1 in the presence of NNM, *C*_WT-His-P9-1_: 20 μM, *C*_NNM_: 1–13: 0, 4.0, 8.0, 12.0, 16.0, 20.0, 24.0, 28.0, 32.0, 36.0, 40.0, 44.0, 48.0 μM. (**C**) Based on microscale themophoresis, WT-His-P9-1 can bind with DFL, with a *K*_d_ of 3.26 μM. (**D**) Based on microscale themophoresis, WT-His-P9-1 can bind with NNM, with a *K*_d_ of 48.40 μM.

The fluorescence emission spectra obtained for WT-His-P9-1 at pH7.4 with the addition of DFL and NNM are shown in Figure 4. The results showed that the fluorescence intensity of WT-His-P9-1 decreased in the presence of DFL and NNM, indicating that the binding of molecules to protein quenched the intrinsic fluorescence of WT-His-P9-1.

The constants of DFL and NNM binding to WT-His-P9-1 are listed in Table 3.

**Table 3:** Stern–Volmer quenching constants, binding parameters, and thermodynamic parameters of the WT-His-P9-1+DFL and WT-His-P9-1+NNM systems.

	Stern–Volmer quenching constants			Binding parameters		
No.	*K*_q_ (M^−1^·S^−1^)	*K*_SV_ (M^−1^)	*R*	*K*_A_ (M^−1^)	*n*	*R*
WT-His-P9-1+DFL	7.817 × 10^10^	7.817 × 10^2^	0.991	1 × 10^5.061^	1.139	0.991
WT-His-P9-1+NNM	1.040 × 10^12^	1.040 × 10^4^	0.986	1 × 10^4.244^	1.053	0.991

The results showed that the *K*_sv_ and *K*_q_ values were greater than the limiting diffusion rate constant of biopolymers (2 × 10^10^ M^−1^·S^−1^), which indicated that the probable quenching of WT-His-P9-1 caused by DFL and NNM follows a static quenching mechanism [36].

The *K*_A_ and *n* values of the WT-His-P9-1+DFL and WT-His-P9-1+NNM systems are summarized in Table 3. Obviously, the WT-His-P9-1+DFL system with a *K*_A_ of 1 × 10^5.061^ (Figure 4A) exhibited a higher binding affinity than the WT-His-P9-1+NNM system with a *K*_A_ of 1 × 10^4.244^ (Figure 4B). The value of binding sites *n* was approximately 1, suggesting that the interactions of WT-His-P9-1 with DFL and NNM likely occurred in one affinity binding site.

To validate the findings in FT, we employed MST [37]. This technology probes for fluorescent changes in the hydration shell of molecules to measure protein—Protein or protein—Small molecule interactions with high sensitivity in near-native conditions. The biochemical binding activity of DFL to WT-His-P9-1 protein with a series of DFL titrations was confirmed using MST, with the titration of NNM to WT-His-P9-1 protein for comparison. DFL showed binding activity to WT-His-P9-1 in the titration assays, yielding a *K*_d_ value of 3.26 μM (Figure 4C). The NNM titration yielded a *K*_d_ value of 48.40 μM (Figure 4D).

### 3.6. Binding Sites of DFL to SRBSDV-P9-1 Mutant Protein

**Figure 5:**
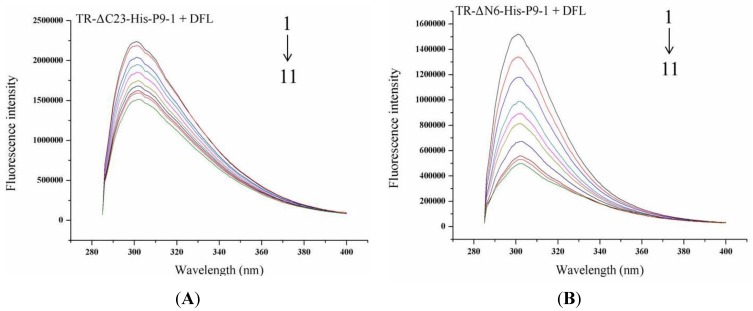

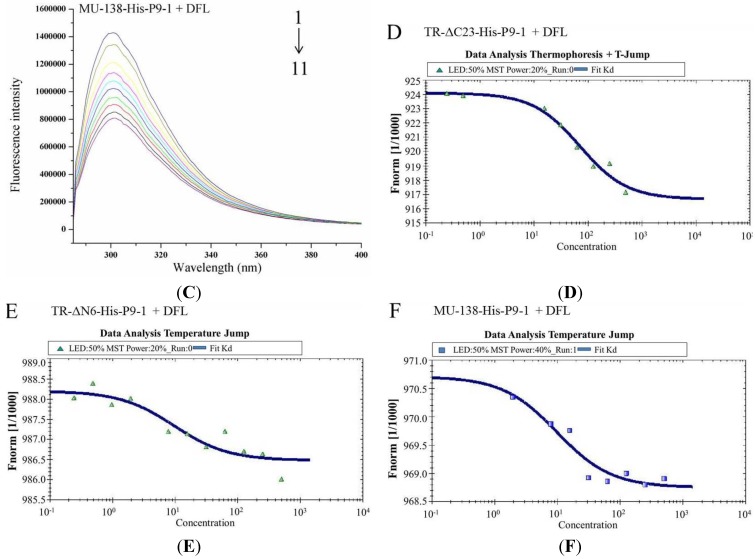
Binding affinity and specificity of DFL to mutagenesis P9-1 proteins. (**A**) Fluorescence emission spectra of TR-ΔC23-His-P9-1 in the presence of DFL, *C*_TR-ΔC23-His-P9-1_: 20 μM, *C*_DFL_: 1–11: 0, 2.0, 4.0, 6.0, 8.0, 10.0, 12.0, 14.0, 16.0, 18.0, 20.0 μM. (**B**) Fluorescence emission spectra of TR-ΔN6-His-P9-1 in the presence of DFL, *C*_TR-ΔN6-His-P9-1_: 20 μM, *C*_DFL_: 1–11: 0, 2.0, 4.0, 6.0, 8.0, 10.0, 12.0, 14.0, 16.0, 18.0, 20.0 μM. (**C**) Fluorescence emission spectra of MU-138-His-P9-1 in the presence of DFL, *C*_MU-138-His-P9-1_: 20 μM, *C*_DFL_: 1–11: 0, 2.0, 4.0, 6.0, 8.0, 10.0, 12.0, 14.0, 16.0, 18.0, 20.0 μM. (**D**) The ability of DFL binding to TR-ΔC23-His-P9-1 protein decreased, with a *K*_d_ of 70.30 μM, based on microscale themophoresis. (**E**) Constitutively active DFL binds to TR-ΔN6-His-P9-1protein, with a *K*_d_ of 9.55 μM, based on microscale themophoresis. (**F**) The experiment described in (**E**) was repeated for DFL with MU-138-His-P9-1 protein, with a *K*_d_ of 9.73 μM.

Three mutagenesis proteins were visually designed to explore the vital amino acid structural domain and investigate the conservative *C* terminus, *N* terminus, and serine on position 138. Based on the analysis of DFL binding to WT-His-P9-1, the *K*_SV_, *K*_q_, *K*_A_, and *n* values of DFL binding to the three SRBSDV-P9-1 mutant proteins were deduced (Table 4, Figure 5A, Figure 5B, Figure 5C). The value of the binding sites *n* was approximately 1, suggesting that the interactions of DFL with the SRBSDV-P9-1 mutant proteins likely occurred in one affinity binding site. The binding affinity of DFL to TR-ΔC23-His-P9-1 decreased from 1 × 10^5.061^ to 1 × 10^4.470^. No change was observed in the binding of DFL to MU-138-His-P9-1, with a *K*_A_ of 1 × 10^5.026^. Interestingly, the binding affinity of DFL to TR-ΔN6-His-P9-1 did not decrease but increased from 1 × 10^5.061^ to 1 × 10^6.421^.

**Table 4:** Stern–Volmer quenching constants, binding parameters, and thermodynamic parameters of DFL to SRBSDV-P9-1 mutant proteins.

	Stern–Volmer quenching constants			Binding parameters		
No.	*K*_q_ (M^−1^·S^−1^)	*K*_SV_ (M^−1^)	*R*	*K*_A_ (M^−1^)	*n*	*R*
TR-ΔC23-His-P9-1+DFL	4.715 × 10^10^	4.715 × 10^2^	0.991	1 × 10^4.470^	1.074	0.992
TR-ΔN6-His-P9-1+DFL	5.751 × 10^11^	5.751 × 10^3^	0.986	1 × 10^6.421^	1.286	0.992
MU-138-His-P9-1+DFL	8.750 × 10^10^	8.750 × 10^2^	0.997	1 × 10^5.026^	1.082	0.990

The MST results indicated that the DFL binding to TR-ΔC23-His-P9-1, TR-ΔN6-His-P9-1, and MU-138-His-P9-1of P9-1 protein yielded *K*_d_ values of 51.40, 8.09, and 9.73 μM, respectively (Figure 5D, Figure 5E, Figure 5F). The mutagenesis of the *C* terminus of 23 amino acids led to a 15.77-fold increase in the *K*_d_ value from 3.26 μM to 51.40 μM. However, the *K*_d_ values of the *N*-terminus and Ser138 residues of P9-1 had almost no change. Therefore, the mutagenesis of the *C* terminus of 23 amino acid residues disrupted the binding ability of DFL to P9-1 protein.

## 4. Discussion

In recent years, plant virus diseases have caused significant economic loss in crop fields. However, control of plant virus disease is very difficult and effective antiviral agents are very limited. DFL is a kind of environmentally friendly antiviral agent, which was discovered by our laboratory. It is highly effective against plant viruses through activating systemic acquired resistance (SAR) in plants [38]. Ten years of field trial investigations in China demonstrated that DFL had favorable antiviral efficacies on preventing and controlling tobacco, vegetable and rice viral diseases. On the other hand, antiviral proteins can also activate plant resistance against viral diseases. As reported, a hot pepper (*Capsicum annuum*) cDNA clone encoding pathogenesis-related protein 10 (CaPR-10) [39], hypersensitive response inducing protein 1 (Hrip1) from necrotrophic fungus, *Alternaria tenuissima* [40], and a novel protein from *Verticillium dahliae* (PevD1) [41] have inhibition activities to tobacco mosaic virus (TMV) infection. Among them, Hrip 1, a novel elicitor that represents a potential pathway for engineering resistance of plants against virus, has favorable control effects on TMV and tomato yellow leaf curl virus disease in field application. Based on its immune activation mechanism, Hrip 1 should also be effective against rice virus diseases. Moreover, besides the immune response activation ability on plants, DFL was also found possessing replication inhibition activities on rice virus, especially SRBSDV [17], which make it widely used in field applications in more than ten provinces in Southern China. However, so far the mechanism of this replication inhibition activity of DFL remains unknown. Based on the vital function of SRBSDV-P9-1, the remarkable potency and selectivity of DFL prompted the examination of the molecular mechanisms of targeting P9-1 protein in more detail. Consequently, in this study, a platform that contains complete sequence analysis, protein expression, and targeting mechanism evaluation system for antiviral molecule such as DFL was established. At the beginning of this study, the complete nucleotide sequences of the SRBSDV-P9 of nine isolates were reported, providing their amino acid composition details. SRBSDV-P9 was 1900 nt in length and contained two ORFs to encode Vps formation protein P9-1 and an unknown protein P9-2. In addition, the sequence organization of GZDYun-P9-1 showed very high aa identity, ranging from 98% to 100% with SRBSDV-P9-1 and from 76% to 78% with RBSDV-P9-1. This high identity could be owned by the genus *Fijivirus*, and P9-1, which is a minimal viral component protein required for Vps formation, performs an important function in virus infection [14]. In the amino acid sequence of RBSDV-P9-1, 41 residues were leucine (11%), and these residues participate in leucine zipper-like interactions with molecules, such that the P9-1 helical octamer bundle was stabilized by these interactions [42]. Similarly, 43 leucine (12%) residues exist in SRBSDV-P9-1 (Supplementary File 1). Thus, the hydrophobic interactions contributed by leucine (12%) residues may also stabilize the SRBSDV-P9-1 helical octamer bundle formation.

The alignment results indicated that P9-1 protein had highly conserved *C*- and *N*-terminal amino acid residues and a hypervariable region that differ from 131 aa to 160 aa compared with RBSDV (Figure 2). To develop a preliminary understanding of the important amino acid structural domain in P9-1 protein, a series of mutagenesis P9-1 proteins, namely, TR-ΔC23-His-P9-1, TR-ΔN6-His-P9-1, and MU-138-His-P9-1 was constructed. Among the mutant proteins, TR-ΔC23-His-P9-1 and TR-ΔN6-His-P9-1 were cloned by deleting 23 conserved *C*-terminal residues and 6 conserved *N*-terminal residues to pET28a, respectively. In the hypervariable region from 131 aa to 160 aa, the SNP of S **→** T^415^ located on the CDS position of Ser138 was found in the site-directed mutagenesis. Simultaneously, three conservative sites (E134, E146, and P150) were found in the 131 aa to 160 aa region of SRBSDV-P9-1 and RBSDV-P9-1. These three conservative sites may be investigated further to confirm the binding ability to SRBSDV-P9-1 protein. Moreover, three kinds of P9-1 mutant proteins were expressed and purified successfully.

To evaluate the binding strength of DFL targeting on P9-1 protein, WT-His-P9-1 protein was purified first, and binding tests were performed by FT and MST, with NNM as control. The FT results indicated that the interactions of WT-His-P9-1 with DFL and NNM likely occurred in one affinity binding site (Table 3). Moreover, the WT-His-P9-1+DFL system with a *K*_A_ of 1 × 10^5.061^ exhibited a higher binding affinity than the WT-His-P9-1+NNM system with a *K*_A_ of 1 × 10^4.244^ (Figure 4A). As predicted in MST, DFL displayed high-affinity binding to WT-His-P9-1 with an approximate *K*_d_ of 3.26 μM. The affinity of WT-His-P9-1 to molecules upon evaluating the binding constant suggested that DFL had the strongest combination to protein P9-1 and was superior to NNM (Figure 4B). The data indicated that DFL could efficiently target WT-His-P9-1 within a 10^5^ range in FT and in the μM range in MST analysis system, thereby becoming superior to NNM. The strong binding affinity of DFL targeting on P9-1 could be the reason for inhibiting SRBSDV replication.

Finally, to discover the binding sites of DFL to WT-His-P9-1, binding assays were conducted between DFL and three different kinds of mutant proteins, namely, TR-ΔN6-His-P9-1, TR-ΔC23-His-P9-1, and MU-138-His-P9-1. In the FT screening platform, TR-ΔC23-His-P9-1 strongly inhibited the combining capacity of DFL with almost a 10-fold decrease in *K*_A_ from 1 × 10^5.061^ to 1 × 10^4.470^. The result from the MST analysis was consistent with that obtained by FT, with a 15.77 fold increase in *K*_d_ value from 3.26 μM to 51.40 μM. However, for TR-ΔN6-His-P9-1, despite the increase in *K*_A_ value up to 1 × 10^6.421^, the *K*_d_ value of 9.55 μM did not change compared with that of WT-His-P9-1. For MU-138-His-P9-1, the *K*_A_ and *K*_d_ values of 1 × 10^5.026^ and 9.73 μM, respectively, did not change compared with that of WT-His-P9-1. Based on the FT and MST test data, the 23 amino acids of the *C* terminal were important to DFL inexerting inhibition ability to SRBSDV replication. As reported, a crystallographic analysis of RBSDV-P9-1 revealed the structural features that allowed the protein to form dimers via hydrophobic interactions, and each dimer has carboxy-terminal regions that resemble arms and extend to neighboring dimers, thereby uniting sets of four dimers via lateral hydrophobic interactions to yield cylindrical octamers, which are vital to the Vps matrix [42]. These reports indicate that the *C*-terminus 23 amino acid residues were specifically vital to the binding affinity.

## 5. Conclusions

In this paper, the mechanism of the action of DFL against SRBSDV in rice was explained. First, the biological sequence information was described. The sequence analysis of nine isolates provided detailed amino acid composition, which indicated that P9-1 protein had highly conserved *C*- and *N*-terminal amino acid residues and a hypervariable region that differ from 131 aa to 160 aa. Second, wild-type (WT-His-P9-1), 23 *C*-terminal residues truncated (TR-ΔC23-His-P9-1), 6 *N*-terminal residues truncated (TR-ΔN6-His-P9-1), and Ser138 site-directed (MU-138-His-P9-1) mutant proteins were expressed. Third, we found that DFL had micromole affinity with SRBSDV P9-1 *in vitro*. Finally, we confirmed that DFL binds with the internal pore of P9-1 octamers, which was important for the functioning of the Vps formations in SRBSDV by binding with the 23 carboxy-terminal residues. To the best of our knowledge, this study was the first to investigate the rational targeting of nonstructural P9-1 protein; a principle that could be viable for finding new antiviral drugs and anti-proliferative mechanisms to SRBSDV was established.

## Supplementary Files

Supplementary File 1
