# Supplementary material for: Interaction Research on the Antiviral Molecule Dufulin Targeting on Southern Rice Black Streaked Dwarf Virus P9-1 Nonstructural Protein
Source: Viruses. 2015 Mar 23;7(3):1454–73. doi: 10.3390/v7031454 (PMC4379580; doi:10.3390/v7031454)
Supplement: Supplementary File 1 [file viruses-07-01454-s001.pdf]

Supplementary Information

Supplementary File 1: Amino acid sequence of SRBSDV-GZDYun-P9-1.

MADLERRTFGSYKIEELTIRNDQPTRNTNLSLSQSTENRLSTKKIPLLDDGIFELLNYLIDGTN FNKTCYCGFNYSHLPNLERDFNIASLYVRENFEICTDQLDLANYVRQPNISIKSPDFTVCLEYV LKTVVESESSTKDQKDDESQKPTSTDSTKNEQEKKFVEMSLLPLLNRESEESLTEEILEGEGAV VNVLKLFIKGFLMHLGENPNSYDRQLTVEKYRPLLVSIVGYEYLVGTTVPEKKINHIYYQLATF DNYPFDLLRFQLSSLISTPTSILERITKEGLFKIITSSTLRGATRQTVLFRGINGSESFLNIKRYRRF RTRIVGNADSVIKSDFSSLKLDV *.

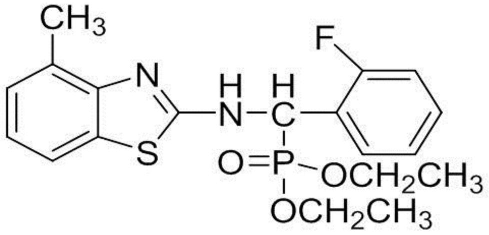

DFL

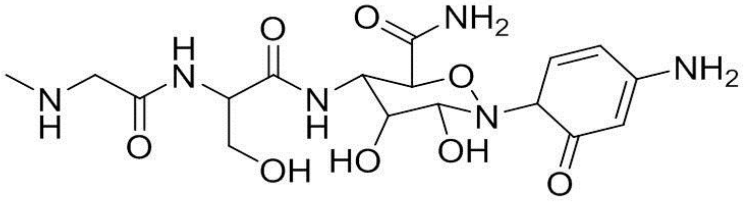

Ningnanmycin

Figure S1. Chemical structures of DFL and NNM.

© 2015 by the authors; licensee MDPI, Basel, Switzerland. This article is an open access article distributed under the terms and conditions of the Creative Commons Attribution license (http://creativecommons.org/licenses/by/4.0/).
